# Atezolizumab Plus Bevacizumab for TACE‐Unsuitable Intermediate‐Stage HCC Beyond Up‐To‐7 Criteria: Final Analysis of REPLACEMENT

**DOI:** 10.1111/liv.70379

**Published:** 2025-11-03

**Authors:** Kazuomi Ueshima, Kaoru Tsuchiya, Tatsuya Yamashita, Shigeo Shimose, Kazushi Numata, Yuzo Kodama, Shinji Itoh, Yasuhito Tanaka, Hidekatsu Kuroda, Kazuyoshi Ohkawa, Teiji Kuzuya, Masafumi Ikeda, Youhei Kooka, Hiroshi Aikata, Atsushi Hiraoka, Michihisa Moriguchi, Ryosuke Tateishi, Sadahisa Ogasawara, Kouji Yamamoto, Masatoshi Kudo

**Affiliations:** ^1^ Department of Gastroenterology and Hepatology Kindai University Faculty of Medicine Osaka Japan; ^2^ Department of Gastroenterology and Hepatology Japanese Red Cross Musashino Hospital Tokyo Japan; ^3^ Department of Gastroenterology Kanazawa University Hospital Ishikawa Japan; ^4^ Division of Gastroenterology, Department of Medicine Kurume University School of Medicine Kurume Japan; ^5^ Gastroenterological Center Yokohama City University Medical Center Yokohama Kanagawa Japan; ^6^ Division of Gastroenterology, Department of Internal Medicine Kobe University Graduate School of Medicine Kobe Japan; ^7^ Department of Surgery and Science, Graduate School of Medical Sciences Kyushu University Fukuoka Japan; ^8^ Department of Gastroenterology and Hepatology, Faculty of Life Sciences Kumamoto University Kumamoto Japan; ^9^ Division of Gastroenterology and Hepatology, Department of Internal Medicine Iwate Medical University Iwate Japan; ^10^ Department of Hepatobiliary and Pancreatic Oncology, OICI ‐ Osaka International Cancer Institute Osaka Japan; ^11^ Department of Gastroenterology and Hepatology Fujita Health University Toyoake Japan; ^12^ Department of Hepatobiliary and Pancreatic Oncology National Cancer Center Hospital East Kashiwa Japan; ^13^ Department of Hepatology Sendai Kousei Hospital Sendai Japan; ^14^ Department of Gastroenterology and Hepatology Hiroshima Prefectural Hospital Hiroshima Japan; ^15^ Department of Gastroenterology Ehime Prefectural Central Hospital Ehime Japan; ^16^ Department of Molecular Gastroenterology and Hepatology, Graduate School of Medical Science Kyoto Prefectural University of Medicine Kyoto Japan; ^17^ Department of Gastroenterology, Graduate School of Medicine The University of Tokyo Tokyo Japan; ^18^ Department of Gastroenterology, Graduate School of Medicine Chiba University Chiba Japan; ^19^ Department of Biostatistics, Yokohama City University Graduate School of Medicine Kanagawa Japan

**Keywords:** atezolizumab, bevacizumab, hepatocellular carcinoma, transcatheter arterial chemoembolization (TACE)

## Abstract

**Background and Aims:**

The phase II REPLACEMENT study showed promising clinical benefit from atezolizumab plus bevacizumab in transcatheter arterial chemoembolization (TACE)–naïve patients with intermediate‐stage hepatocellular carcinoma (HCC) beyond up‐to‐7 criteria, meeting its primary endpoint of progression‐free survival (PFS). Here, we report the final overall survival (OS) analysis.

**Methods:**

Enrolled patients were naïve to TACE with unresectable intermediate‐stage HCC beyond up‐to‐7 criteria, had Child‐Pugh A, Eastern Cooperative Oncology Group performance status 0/1 and received no previous systemic therapy. Atezolizumab 1200 mg and bevacizumab 15 mg/kg were administered every 3 weeks. The primary endpoint was the 6‐month PFS rate by modified Response Evaluation Criteria in Solid Tumours for HCC (mRECIST); secondary endpoints included OS, PFS by RECIST version 1.1, objective response rate (ORR) and safety.

**Results:**

Overall, 74 patients were enrolled between December 2020 and September 2021. At the clinical cut‐off date (March 31, 2024), median follow‐up was 33.6 months. Median PFS by mRECIST was 9.1 months (95% CI 7.1–10.2). Median OS was 33.8 months (95% CI 22.6–not estimable). ORR was 40.5% (95% CI 29.3–52.6), with 12.2% of patients having a complete response. Overall, 82.4% of patients received subsequent therapy. All‐cause adverse events (AEs) were observed in 98.6% of patients, most commonly hypertension (71.6%) and proteinuria (54.1%). Grade 3/4 AEs occurred in 43.2% of patients; no Grade 5 AEs were reported.

**Conclusions:**

These results show that atezolizumab plus bevacizumab can be an alternative treatment option for patients with intermediate‐stage HCC beyond up‐to‐7 criteria who are deemed unsuitable for TACE.

**Trial Registration:**

jRCTs071200051

**Summary**

- The phase 2 REPLACEMENT trial evaluated atezolizumab plus bevacizumab as a first‐line treatment for patients with unresectable hepatocellular carcinoma who were not suitable for transcatheter arterial chemoembolization (TACE).
- The final overall survival data and acceptable safety profile, supported by the previously reported progression‐free survival data, suggest a benefit from this regimen.
- Atezolizumab plus bevacizumab could be helpful for patients who are not suitable for the standard TACE treatment.

**Abbreviations**
AE: adverse event
ALBI: albumin‐bilirubin
BCLC: Barcelona Clinic Liver Cancer
CI: confidence interval
CR: complete response
CTCAE: Common Terminology Criteria for Adverse Events
ECOG: Eastern Cooperative Oncology Group
FAS: full analysis set
HCC: hepatocellular carcinoma
mRECIST: modified Response Evaluation Criteria in Solid Tumours
ORR: objective response rate
OS: overall survival
PFS: progression‐free survival
PR: partial response
RECIST 1.1: Response Evaluation Criteria in Solid Tumours version 1.1
TACE: transcatheter arterial chemoembolization

## 1 Introduction

Transcatheter arterial chemoembolization (TACE) was developed as the standard of care for patients with intermediate‐stage hepatocellular carcinoma (HCC) at a time when systemic therapy was unavailable. Recent consensus guidelines recommend systemic therapy for patients who are not suitable for TACE, with the goal of improving overall survival (OS) and preserving liver function [1, 2]. The phase III IMbrave150 study established atezolizumab plus bevacizumab as a standard of care in patients with unresectable HCC [3].

REPLACEMENT was a multicentre, phase II study evaluating the efficacy and safety of atezolizumab plus bevacizumab in TACE‐naïve patients with intermediate‐stage HCC beyond up‐to‐7 criteria [4]. In the previously reported primary analysis of the study, a 6‐month progression‐free survival (PFS) rate of 66.8% (90% confidence interval [CI] 56.8–75.0) was observed. The lower limit of the 90% CI for the 6‐month PFS rate exceeded the prespecified threshold of 55%, indicating that the primary endpoint was met [4]. However, at the time of the primary analysis, with a median follow‐up of 15.1 months, an interim analysis of OS found that the data were immature. Here, we report the final OS analysis of the REPLACEMENT trial with an additional 18.5 months of follow‐up from the primary analysis.

## 2 Materials and Methods

### 2.1 Study Design, Participants and Treatment

This was a prospective, multicentre, single‐arm, phase II clinical study conducted at 35 sites in Japan (jRCTs071200051). This study was conducted in accordance with the Declaration of Helsinki, the Japanese Clinical Trials Act, the Ordinance for Enforcement of the Clinical Trials Act of the Ministry of Health, Labour and Welfare and related ethical guidance. Patients were enrolled after giving consent to participate using a written informed consent form, which was approved by the Certified Review Board of Kyushu University.

Inclusion and exclusion criteria have been previously described [4]. Briefly, patients had unresectable intermediate‐stage HCC beyond up‐to‐7 criteria [2, 5], Child‐Pugh class A and Eastern Cooperative Oncology Group (ECOG) performance status of 0 or 1. Patients had no previous systemic therapy or TACE treatment, excluding TACE as part of a regimen with ablation or surgery.

Enrolled patients received atezolizumab 1200 mg and bevacizumab 15 mg/kg every 3 weeks, consistent with the respective package inserts, until discontinuation due to disease progression, adverse events (AEs) or other reasons.

### 2.2 Outcomes and Assessments

The previously reported [4] primary endpoint was the 6‐month PFS rate according to the modified Response Evaluation Criteria in Solid Tumours (mRECIST) for HCC [6] by investigator assessment. PFS was defined as the period from the enrolment date to disease progression or death from any cause, whichever occurred first. The study was considered positive if the lower bound of the two‐sided 90% CI of the 6‐month PFS for atezolizumab plus bevacizumab was > 55%.

Secondary endpoints included PFS according to Response Evaluation Criteria in Solid Tumours version 1.1 (RECIST 1.1) [7] by investigator assessment; OS, defined as the period from the enrolment date to the date of death due to any cause; objective response rate (ORR), defined as the percentage of patients with the best overall response of complete response (CR) or partial response (PR), according to mRECIST and RECIST 1.1 by investigator assessment. We also evaluated ORR including responses after subsequent curative intent therapy, which was defined as resection, radiofrequency ablation (RFA) or TACE with curative intent, irrespective of the actual outcome, performed after tumour shrinkage in response to atezolizumab plus bevacizumab. Safety was also assessed as a secondary endpoint.

### 2.3 Statistical Analysis

The full analysis set (FAS) consisted of all enrolled patients (intention‐to‐treat population) excluding ineligible patients and those who withdrew consent or did not receive protocol treatment. The FAS was used as the primary analysis population for all efficacy endpoints.

Exploratory subgroup analyses were conducted to explore the association between tumour response and OS, by comparing OS among patients in the FAS with measurable lesions at baseline, categorised by their best overall response: CR/PR, stable disease (SD) or progressive disease (PD). Additionally, to assess the impact of subsequent curative intent therapy, OS was compared between patients who received curative intent therapy (resection, RFA or TACE with curative intent) and those who did not within the FAS. This comparison was then expanded by stratifying patients who did not receive curative intent therapy by their best overall response (CR/PR, SD or PD). To mitigate potential immortal time bias in these exploratory analyses, we performed a landmark analysis for OS at 6 months, limiting the analysis to patients who survived from enrolment to that landmark time point.

For the primary endpoint of PFS, the Kaplan–Meier method was used to estimate PFS, and the 90% CI for the 6‐month PFS rate was calculated using Greenwood's formula, while the 95% CI for the median PFS was determined using the Brookmeyer–Crowley method. OS was analysed using the Kaplan–Meier method. Hazard ratios (HRs) were estimated using a Cox proportional hazards model.

The safety analysis set (safety population) consisted of all enrolled patients who began protocol treatment and from whom at least some data were obtained. The frequency and severity of AEs were assessed according to Common Terminology Criteria for Adverse Events (CTCAE) version 5.0. The time course of albumin‐bilirubin (ALBI) and Child‐Pugh scores was assessed by calculation of means and standard deviations in each treatment cycle. SAS version 9.4 (SAS Institute Inc., Cary, NC, USA) was used for all statistical analyses.

## 3 Results

### 3.1 Patient Characteristics

Overall, 74 patients were enrolled between December 2020 and September 2021 (clinical cutoff: March 31, 2024; median follow‐up, 33.6 months). The baseline characteristics have been previously described [4].

### 3.2 Efficacy

#### 3.2.1 Primary and Secondary Analyses

**FIGURE 1:**
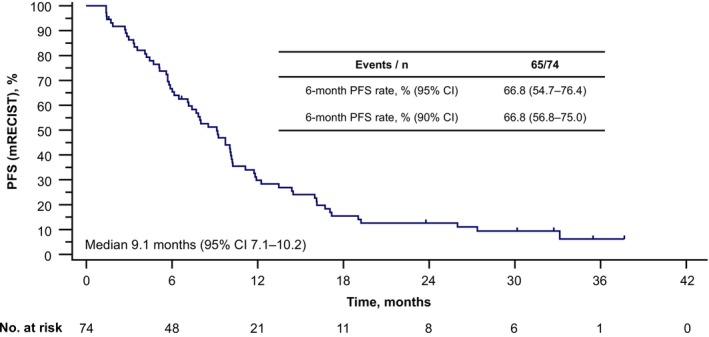
Progression‐free survival by mRECIST. CI, confidence interval; mRECIST, modified Response Evaluation Criteria in Solid Tumours; PFS, progression‐free survival.

**FIGURE 2:**
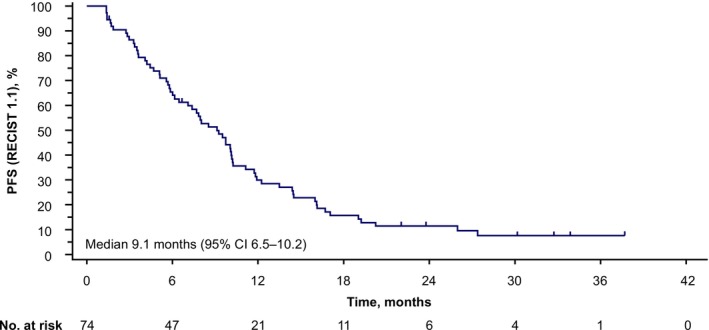
Progression‐free survival by RECIST 1.1. CI, confidence interval; RECIST 1.1, Response Evaluation Criteria in Solid Tumours version 1.1; PFS, progression‐free survival.

Among the 74 patients in the FAS, 65 (87.8%) had disease progression by mRECIST during the follow‐up period (Figure 1). Median PFS by mRECIST was maintained from the primary analysis at 9.1 months (95% CI 7.1–10.2). Median PFS by RECIST 1.1 was 9.1 months (95% CI 6.5–10.2) (Figure 2).

**FIGURE 3:**
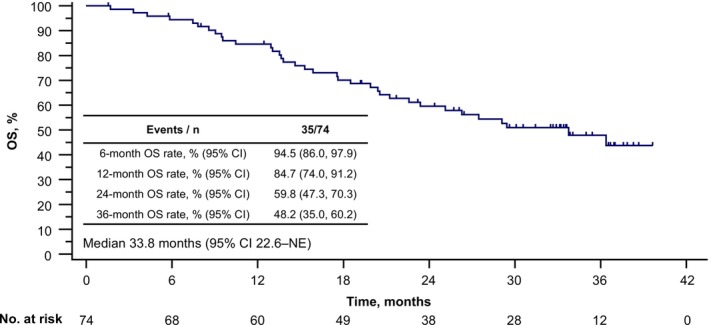
Overall survival. CI, confidence interval; NE, not estimable; OS, overall survival.

Overall, 35 patients (47.3%) had died in the follow‐up period, with a median OS of 33.8 months (95% CI 22.6–not estimable) (Figure 3). The 6‐, 12‐, 24‐ and 36‐month OS rates were 94.5% (95% CI 86.0–97.9), 84.7% (95% CI 74.0–91.2), 59.8% (95% CI 47.3–70.3) and 48.2% (95% CI 35.0–60.2), respectively.

The ORR assessed by mRECIST was 40.5% (95% CI 29.3–52.6), with 12.2% of patients having CR (Table 1). The ORR assessed by mRECIST including responses after curative‐intent therapy was 44.6% (95% CI 33.0–56.6), with 17.6% of patients having CR.

**TABLE 1:** Best overall response.

Best overall response	mRECIST	mRECIST^a^	RECIST 1.1	RECIST 1.1^a^
ORR, *n* (%) [95% CI]	30 (40.5) [29.3–52.6]	33 (44.6) [33.0–56.6]	18 (24.3) [15.1–35.7][95% CI]	23 (31.1) [20.8–42.9]
CR, *n* (%)	9 (12.2)	13 (17.6)	0 (0.0)	5 (6.8)
PR, *n* (%)	21 (28.4)	20 (27.0)	18 (24.3)	18 (24.3)
SD, *n* (%)	37 (50.0)	34 (45.9)	48 (64.9)	43 (58.1)
PD, *n* (%)	5 (6.8)	5 (6.8)	6 (8.1)	6 (8.1)
NE, *n* (%)	2 (2.7)	2 (2.7)	2 (2.7)	2 (2.7)
DCR, *n* (%) [95% CI]	67 (90.5) [81.5–96.1]	67 (90.5) [81.5–96.1]	66 (89.2) [79.8–95.2]	66 (89.2) [79.8–95.2]

Abbreviations: CI, confidence interval; CR, complete response; DCR, disease control rate; NE, not evaluable; ORR, objective response rate; PD, progressive disease; PR, partial response; SD, stable disease.

^a^ Including responses after curative intent therapy.

#### 3.2.2 Exploratory Analyses

To assess the association between tumour response per mRECIST and OS, OS was compared between patients who achieved a CR or PR and those who did not (SD or PD). Baseline characteristics by response category are shown in Table S1. At baseline, patients in the CR/PR group were more likely to have ALBI Grade 2. The tumour burden in this group also tended to be less, as characterised by a smaller maximum tumour diameter, a reduced sum of longest diameters and fewer tumours. Consequently, the proportion of patients with tumour burden beyond the up‐to‐11 criteria was also lower in this group.

OS was numerically longer among patients who achieved CR/PR than those with SD or PD (Figure S1). Compared with the PD group, the HRs for OS were 0.16 (95% CI 0.04–0.59) for the CR/PR group and 0.54 (95% CI 0.16–1.81) for the SD group. This finding was consistent with the results of the 6‐month landmark analysis.

Ten patients (13.5%) underwent subsequent curative intent therapy, 2 (2.7%) received resection or RFA, and 8 (10.8%) received TACE with curative intent (Table S2). At baseline, patients who received curative intent therapy had a lower proportion of prior surgery or ablation but tended to have a greater tumour burden, with a larger maximum tumour diameter, a larger sum of longest diameters and a higher proportion of patients with ≥ 6 tumours. Regarding the best response to prior atezolizumab plus bevacizumab, a higher proportion of patients who received curative intent therapy achieved CR or PR (Table S2).

Subsequent curative intent therapy was associated with longer OS versus no curative intent therapy (Figure S2). When compared with patients who did not receive curative intent therapy (*n* = 64), the HR was not estimable for those who underwent resection or RFA (*n* = 2) due to the absence of events in this subgroup, and the HR was 0.63 (95% CI 0.19–2.05) for patients who received TACE with curative intent (*n* = 8). This finding was consistent with the 6‐month landmark analysis, which showed that the HR was not estimable for resection or RFA, while the HR for TACE with curative intent was 0.70 (95% CI, 0.21–2.33).

OS analyses among patients who received curative intent therapy versus subgroups of patients who did not, defined by their best overall response, are shown in Figure S3. Compared with the group of patients with PD who did not receive curative intent therapy, the HRs for OS were not estimable for patients who received resection or RFA, 0.25 (95% CI, 0.05–1.24) for those who received TACE with curative intent, 0.18 (95% CI, 0.05–0.70) for the CR/PR subgroup, and 0.54 (95% CI, 0.16–1.83) for the SD subgroup among patients who did not receive curative intent therapy. These results were consistent with the 6‐month landmark analysis, which showed corresponding HRs of not estimable, 0.34 (95% CI, 0.06–2.04), 0.17 (95% CI, 0.03–0.90) and 0.76 (95% CI, 0.18–3.27), respectively.

### 3.3 Subsequent Therapies

Overall, 61 patients (82.4%) received subsequent therapy, including 48 (64.9%) who received systemic therapy and 31 (41.9%) who received TACE (Table 2). The most common subsequent systemic therapies, administered as second‐ or later‐line therapy, were lenvatinib (received by 32 patients [43.2%]), durvalumab plus tremelimumab (received by 13 patients [17.6%]) and atezolizumab plus bevacizumab (received by 12 patients [16.2%] as a third‐ or later‐line therapy).

**TABLE 2:** Subsequent therapies.

Subsequent therapy, *n* (%)	*n* = 74
Any	61 (82.4)
Systemic therapy	48 (64.9)
Lenvatinib	32 (43.2)
Durvalumab + tremelimumab	13 (17.6)
Atezolizumab + bevacizumab	12 (16.2)
Cabozantinib	8 (10.8)
Regorafenib	7 (9.5)
Sorafenib	5 (6.8)
Ramucirumab	4 (5.4)
Investigational antineoplastic agents	4 (5.4)
TACE	31 (41.9)
RFA or MWA	10 (13.5)
HAIC	9 (12.2)
Surgery	4 (5.4)
Radiotherapy	4 (5.4)
Other	2 (2.7)

Abbreviations: HAIC, hepatic artery infusion chemotherapy; MWA, microwave ablation; RFA, radiofrequency ablation; TACE, transcatheter arterial chemoembolization.

### 3.4 Safety

All‐cause AEs were observed in 73 patients (98.6%). AEs of any grade reported in ≥ 20% of patients included hypertension in 53 patients (71.6%), proteinuria in 40 (54.1%), malaise in 21 (28.4%) and anorexia in 15 (20.3%) (Table 3). Grade 3/4 AEs occurred in 32 patients (43.2%); Grade 4 AEs were reported in two patients, one with creatine phosphokinase increase and one with sepsis. No Grade 5 AEs were reported.

**TABLE 3:** Most common adverse events.

Safety population (*n* = 74)	AE		Treatment‐related AE	
	Any grade	Grade 3/4	Any grade	Grade 3/4
All AEs, *n* (%)	73 (98.6)	32 (43.2)	69 (93.2)	25 (33.8)
Hypertension	53 (71.6)	14 (18.9)	50 (67.6)	14 (18.9)
Proteinuria	40 (54.1)	8 (10.8)	38 (51.4)	8 (10.8)
Malaise	21 (28.4)	1 (1.4)	17 (23.0)	1 (1.4)
Anorexia	15 (20.3)	1 (1.4)	12 (16.2)	0
Edema	13 (17.6)	0	10 (13.5)	0
Pruritus	12 (16.2)	0	12 (16.2)	0
Diarrhoea	10 (13.5)	1 (1.4)	8 (10.8)	1 (1.4)

Abbreviation: AE, adverse event.

Treatment‐related AEs of any grade occurred in 69 patients (93.2%) and Grade 3/4 treatment‐related AEs occurred in 25 patients (33.8%). The most common Grade 3/4 treatment‐related AEs were hypertension (18.9%) and proteinuria (10.8%).

AEs requiring steroid treatment occurred in 10 patients (13.5%) and included pruritus, adrenal insufficiency, malaise, hypertension, pneumonitis and oral mucositis (Table 4). There were two Grade 3 AEs requiring steroid treatment, one event of adrenal insufficiency and one of pneumonitis.

**TABLE 4:** Adverse events requiring corticosteroids.

Safety population (*n* = 74)	Any grade	Grade 3/4
All AEs requiring corticosteroids, *n* (%)	10 (13.5)	2 (2.7)
Pruritus	3 (4.1)	0
Adrenal insufficiency	2 (2.7)	1 (1.4)
Malaise	2 (2.7)	0
Hypertension	2 (2.7)	0
Pneumonitis	1 (1.4)	1 (1.4)
Oral mucositis	1 (1.4)	0

Abbreviation: AE, adverse event.

**FIGURE 4:**
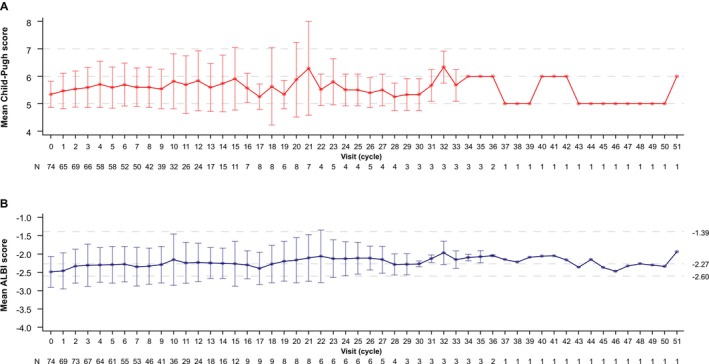
Mean (A) Child‐Pugh and (B) ALBI scores over time. The numbers to the right of the ALBI score graph represent the grade level cutoffs (ALBI Grade 1: ≤ −2.60; modified ALBI Grade 2a: > −2.60 to ≤ −2.27; modified ALBI Grade 2b: > −2.27 to ≤ −1.39; ALBI Grade 3 > −1.39). ALBI, albumin‐bilirubin.

Mean Child‐Pugh scores and mean ALBI scores are shown in Figure 4A,B. Values for both measures remained relatively stable from the first assessment through cycle 15, the points for which ≥ 10 patients were evaluable.

## 4 Discussion

This was a prospective study to evaluate the efficacy and safety of atezolizumab plus bevacizumab for patients with unresectable intermediate‐stage HCC beyond up‐to‐7 criteria who were naïve to TACE. With a median follow‐up of 33.6 months, PFS and ORR results were maintained from the primary analysis results, and the median OS was 33.8 months. The safety profile observed in the updated analysis was consistent with that from the primary analysis. Furthermore, the rate of AEs requiring steroid treatment in this study, which occurred in 13.5% of patients, was similar to the rate observed in the IMbrave150 trial, which was 12.2% in the atezolizumab plus bevacizumab arm [8].

The OS results observed in this study compare favourably to those of other trials evaluating systemic therapy for HCC. In the updated analysis of the IMbrave150 trial, with a median 15.6 months of follow‐up, OS was 19.2 months (95% CI 17.0–23.7) in the intention‐to‐treat population [9] and 25.8 months (95% CI 23.9–not reached) in the subgroup of patients classified as BCLC stage B [10]. Similarly, the SHARP trial reported a median OS of 14.5 months in patients with BCLC B treated with sorafenib (HR vs. placebo 0.72; 95% CI 0.38–1.38) [11]. Among patients with BCLC B in the REFLECT trial, median OS of 18.5 months was observed with lenvatinib and 17.3 months with sorafenib (HR 0.91; 95% CI 0.65–1.28) [12]. Furthermore, Kobayashi et al. reported a median OS of 17.0 months (95% CI 15.3–19.2) with lenvatinib in patients with BCLC stage B2 (Bolondi classification), which corresponds closely to the population in the current study [13]. The favourable median OS in this study suggests that atezolizumab plus bevacizumab is effective as a systemic therapy for unresectable intermediate‐stage HCC beyond up‐to‐7 criteria.

Potential explanations for the long OS observed with atezolizumab plus bevacizumab in this study include the tolerability profile, allowing patients to remain on treatment longer, and the ease of transition to subsequent therapies, allowing patients to receive effective subsequent treatment. In this study, 82.4% of patients transitioned from study treatment to subsequent therapies. In contrast, previous studies, some of which included patients with BCLC stage C, have reported transition rates from 34.0% to 55.6% for atezolizumab plus bevacizumab and 43.1% to 50.7% for lenvatinib [9, 12, 14, 15, 16, 17]. These results support the hypothesis that the high rate of transition to subsequent therapies contributes to improved prognosis, consistent with other studies [18, 19]. Furthermore, achieving an objective response has been reported as an independent predictor of longer OS [20], and a positive correlation between the ORR and OS has also been reported [21]. The high ORR in this study is likely to have contributed to prolonged OS. In fact, our exploratory subgroup analysis revealed that patients who achieved a CR/PR had numerically longer OS compared with those who had SD or PD. This finding supports the association between objective response and longer OS, which is consistent with previous reports [20]. However, it is imperative to interpret these findings with caution. Direct cross‐trial comparisons are inherently flawed due to factors such as differences in study designs, eligibility criteria (e.g., this study specifically enrolled TACE‐naïve patients with unresectable HCC beyond the up‐to‐7 criteria), patient characteristics and the available options and accessibility of subsequent therapies, which differ substantially depending on the study period and country. Additionally, the lack of reported median OS for patients with BCLC stage B treated with immunotherapies other than atezolizumab plus bevacizumab limits the ability to draw definitive conclusions.

TACE has been reported to have an inverse association with tumour burden, with OS shortening as tumour burden increases. Kim et al. reported a median OS of 51.5 months among patients with BCLC stage B1 (Bolondi classification), but an OS of 26, 14.8 and 25 months for those with BCLC B2, B3 and B4, respectively [22]. Biolato et al. reported similar results, with a median OS of 33.0, 20.8, 16.1 and 22.2 months for BCLC B1 to B4, respectively [23]. Hung et al. reported a median OS of 33.1 months for patients within up‐to‐7 criteria, 22.3 months for those beyond up‐to‐7 but within up‐to‐11 criteria and 11.9 months for those beyond up‐to‐11 criteria [24]. They also reported that patients with higher tumour burden had a significantly higher risk of liver function deterioration [24]. In another report, among patients treated with TACE, tumour burden beyond up‐to‐7 criteria was shown to be associated with the deterioration of liver function after treatment [25]. According to Kinki criteria, the time to untreatable progression with TACE was shorter in patients with BCLC stage B2 than in those with stage B1 [26]. Moreover, effectiveness decreased with repeated TACE, suggesting that TACE in patients with high tumour burden may impair liver function without achieving the expected therapeutic effect [26, 27]. In contrast, this study demonstrated that treatment with atezolizumab plus bevacizumab helped to maintain liver function, as assessed by Child‐Pugh and ALBI scores, which could have contributed to prolonged OS.

A notable finding from our exploratory analysis is the potential for conversion to curative intent therapy after tumour downsizing with atezolizumab plus bevacizumab. Although these subgroup analyses should be interpreted with caution due to the small sample sizes, patients who underwent subsequent curative intent therapy (*n* = 10, 13.5%) appeared to have numerically favourable OS versus those who did not. Within this cohort, the trend towards better OS among patients who underwent resection/RFA (*n* = 2) versus those who received TACE with curative intent (*n* = 8) is likely attributable to differences in the responses to both initial systemic therapy and the subsequent curative procedures. Patients who underwent resection/RFA had a more favourable response to the initial systemic therapy and subsequently achieved a CR following their curative procedures. In contrast, the patients who received TACE with curative intent initially consisted of three with SD and five with PR; following the procedure, three of these patients achieved CR while five achieved PR. Importantly, patients who received TACE with curative intent achieved an OS comparable with that of patients who had CR/PR with systemic therapy alone and did not undergo curative therapy. This finding is noteworthy given that the TACE with curative intent group included patients with SD—a response category typically associated with poorer prognosis than CR or PR. The achievement of comparable OS despite the inclusion of patients with SD suggests a potential benefit of sequential TACE with curative intent, even in patients who only achieve SD with initial systemic therapy.

The fact that not all patients achieved CR after TACE with curative intent can be attributed to several factors. First, TACE inherently cannot guarantee CR prospectively; achievement of CR can only be confirmed retrospectively through post‐treatment imaging assessment. Second, in this study, ‘curative intent’ was defined at the discretion of individual investigators, introducing potential variability in patient selection. Third, procedural differences in TACE techniques may have contributed to the incomplete responses observed in some patients.

This study's limitations include its single‐arm design with no comparator arm. Additionally, only patients naïve to TACE were included in the study, and the results may not be translatable to other patient populations. Lastly, the lack of quality‐of‐life assessments limits our ability to assess the comprehensive impact of treatment on the patient experience.

This final OS analysis of the phase II REPLACEMENT study showed that atezolizumab plus bevacizumab can be an alternative treatment option for patients with intermediate‐stage HCC beyond up‐to‐7 criteria who are deemed unsuitable for treatment with TACE.

## Ethics Statement

The study was conducted in accordance with the Declaration of Helsinki, the Japanese Clinical Trials Act, the Ordinance for Enforcement of the Clinical Trials Act of the Ministry of Health, Labour and Welfare and related ethical guidance.

## Consent

The study protocol and written information for patients were approved by the Certified Review Board (CRB) of Kyushu University. This approval, including the informed consent form, was applicable to all participating sites. The full list of participating sites can be found at the Japan Registry for Clinical Trials (jRCTs071200051). All participants provided written informed consent using the CRB‐approved form.

## Conflicts of Interest

**Kazuomi Ueshima** has received support for the current manuscript from Chugai Pharmaceutical Co. Ltd.; grants for research from Chugai Pharmaceutical Co. Ltd.; consulting fees from Chugai Pharmaceutical Co. Ltd., Eisai Co. Ltd., AstraZeneca K.K., Pfizer, Takeda Pharmaceutical Co. Ltd., Eli Lilly Japan K.K. and Bayer Yakuhin Ltd.; honoraria for lectures from Chugai Pharmaceutical Co. Ltd., Takeda Pharmaceutical Co. Ltd., Merck Sharp and Dohme, Sumitomo Pharma Co. Ltd., EA Pharma Co. Ltd., ASKA Pharmaceutical Co. Ltd., Nippon Kayaku Co. Ltd., Otsuka Pharmaceutical Co. Ltd., Eisai Co. Ltd., Eli Lilly Japan K.K., AstraZeneca K.K., Taiho Pharmaceutical Co. Ltd., AbbVie GK, Bayer Yakuhin Ltd., Boston Scientific Japan K.K. and Kowa Company Ltd.; support for attending meetings and/or travel from Chugai Pharmaceutical Co. Ltd.

**Kaoru Tsuchiya** has received honoraria for lectures from Chugai Pharmaceutical Co. Ltd., Eisai Co. Ltd., Takeda Pharmaceutical Co. Ltd. and AstraZeneca K.K.

**Tatsuya Yamashita** has received honoraria for lectures from Chugai Pharmaceutical Co. Ltd., AstraZeneca K.K. and Eisai Co. Ltd.

**Shigeo Shimose** has received honoraria for lectures from AstraZeneca K.K., Chugai Pharmaceutical Co. Ltd. and Eisai Co. Ltd.

**Kazushi Numata** has no conflicts to report.

**Yuzo Kodama** has no conflicts to report.

**Shinji Itoh** has received honoraria for lectures from Chugai Pharmaceutical Co. Ltd., AstraZeneca K.K., Eisai Co. Ltd. and MSD.

**Yasuhito Tanaka** has received scholarship donations from AbbVie GK and Otsuka Pharmaceutical Co. Ltd.; research funding from AbbVie GK, FUJIREBIO Inc., Sysmex Corp., GlaxoSmithKline PLC, Gilead Sciences Inc. and Janssen Pharmaceutical K.K.; and lecture fees from AbbVie GK, Gilead Sciences Inc., Chugai Pharmaceutical Co. Ltd., ASKA Pharmaceutical Holdings Co. Ltd., Otsuka Pharmaceutical Co. Ltd., Takeda Pharmaceutical Co. Ltd., GlaxoSmithKline PLC, AstraZeneca K.K., Eisai Co. Ltd. and HU Frontier.

**Hidekatsu Kuroda** has received honoraria for lectures from Eisai Co. Ltd. and Chugai Pharmaceutical Co. Ltd.

**Kazuyoshi Ohkawa** has received honoraria for lectures from Chugai Pharmaceutical Co. Ltd., AstraZeneca K.K., Eisai Co. Ltd., Gilead Sciences Inc. and Incyte Co., and grants for research from Sumitomo Chemical.

**Teiji Kuzuya** has received speaker's fees from Eisai Co. Ltd., Eli Lilly Japan, Chugai Pharmaceutical Co. Ltd. and AstraZeneca.

**Masafumi Ikeda** has received research funding paid to his institution from AbbVie, AstraZeneca K.K., Bayer, Bristol Myers Squibb, Chugai Pharmaceutical Co. Ltd., Eisai Co. Ltd. and MSD. He has also served in a consulting or advisory role for AbbVie, AstraZeneca K.K., Bayer, Chugai Pharmaceutical Co. Ltd., Eisai Co. Ltd., Eli Lilly Japan, MSD and Ono Pharmaceutical Co. Ltd.; received honoraria from Abbott, AstraZeneca, Bristol Myers Squibb, Chugai Pharmaceutical Co. Ltd., Eisai Co. Ltd., Eli Lilly Japan, MSD and Takeda Pharmaceutical Co. Ltd.

**Youhei Kooka** has no conflicts to report.

**Hiroshi Aikata** has received honoraria for lectures from Chugai Pharmaceutical Co. Ltd.

**Atsushi Hiraoka** has received honoraria for lectures from Chugai Pharmaceutical Co. Ltd., Lilly, AstraZeneca K.K., Bayer, Eisai Co. Ltd. and Takeda Pharmaceutical Co. Ltd.

**Michihisa Moriguchi** has received consulting fees from Chugai Pharmaceutical Co. Ltd., and AstraZeneca K.K.; and honoraria for lectures from Chugai Pharmaceutical Co. Ltd., Eisai Co. Ltd., and AstraZeneca K.K.

**Ryosuke Tateishi** has received honoraria for lectures from Chugai Pharmaceutical Co. Ltd., AstraZeneca K.K. and Eisai Co. Ltd.

**Sadahisa Ogasawara** has received grants for research from Eisai Co. Ltd., Chugai Pharmaceutical Co. Ltd., Gilead Sciences, AstraZeneca K.K., Bayer and Eli Lilly; consulting fees from MSD, AstraZeneca K.K. and Chugai Pharmaceutical Co. Ltd.; honoraria for lectures from Eisai, Chugai Pharmaceutical Co. Ltd., Gilead Sciences, Takeda, AstraZeneca K.K., Bayer, Eli Lilly and AbbVie; and participated on advisory boards for MSD, AstraZeneca K.K. and Chugai Pharmaceutical Co. Ltd.

**Kouji Yamamoto** has received research funding from Otsuka Pharmaceutical Co. Ltd., Chugai Pharmaceutical Co. Ltd., Kyowa Kirin Co. Ltd., Euro Doctor Concierge LLC and Daiichi Sankyo Co. Ltd.; consulting fees from Sysmex Corporation and Delta‐Fly Pharma; and honoraria for lectures from FUJIFILM Toyama Chemical Co. Ltd., CMIC CO. Ltd., Daiichi Sankyo Co. Ltd., TME Therapeutics Inc., Takeda Pharmaceutical Co. Ltd., CM Plus Corporation, and AstraZeneca K.K.

**Masatoshi Kudo** has received grants for research from Otsuka Pharmaceutical Co. Ltd., Chugai Pharmaceutical Co. Ltd., Taiho Pharmaceutical Co. Ltd., GE Healthcare Japan Corporation, Eisai Co. Ltd., and AbbVie GK; consulting fees from Chugai Pharmaceutical Co. Ltd., F. Hoffmann‐La Roche Ltd., Eisai Co. Ltd., and AstraZeneca K.K.; and honoraria for lectures from Chugai Pharmaceutical Co. Ltd., Eisai Co. Ltd., AstraZeneca K.K., Eli Lilly Japan K.K., and Takeda Pharmaceutical Co. Ltd.

## Supporting information


**Table S1:** Baseline demographic and clinical characteristics according to best overall response per mRECIST.
**Table S2:** Baseline demographic and clinical characteristics with and without curative intent therapy.
**Figure S1:** Overall survival in patients with objective response (CR/PR) versus SD versus PD per mRECIST (A) without landmarks and (B) with 6‐month landmarks.
**Figure S2:** Overall survival in patients who received subsequent curative intent therapy versus those who did not (A) without landmarks and (B) with 6‐month landmarks.
**Figure S3:** Overall survival in patients who received subsequent curative intent therapy versus those who did not, stratified by best overall response (A) without landmarks and (B) with 6‐month landmarks.

## Data Availability

The data that support the findings of this study are available on request from the corresponding author. The data are not publicly available due to privacy or ethical restrictions.
