# Supplementary material for: Atezolizumab Plus Bevacizumab for TACE‐Unsuitable Intermediate‐Stage HCC Beyond Up‐To‐7 Criteria: Final Analysis of REPLACEMENT
Source: Liver Int. 2025 Nov 3;45(12):e70379. doi: 10.1111/liv.70379 (PMC12580928; doi:10.1111/liv.70379)
Supplement: Supplementary file 1 — Table S1: Baseline demographic and clinical characteristics according to best overall response per mRECIST. Table S2: Baseline demographic and clinical characteristics with and without curative intent therapy. Figure S1: Overall survival in patients with objective response (CR/PR) versus SD versus PD per mRECIST (A) without landmarks and (B) with 6‐month landmarks. Figure S2: Overall survival in patients who received subsequent curative intent therapy versus those who did not (A) without landmarks and (B) with 6‐month landmarks. Figure S3: Overall survival in patients who received subsequent curative intent therapy versus those who did not, stratified by best overall response (A) without landmarks and (B) with 6‐month landmarks. [file LIV-45-0-s001.docx]

**Supplementary Information**

**Supplementary Table S1.** Baseline demographic and clinical characteristics according to best overall response per mRECIST.

| Baseline characteristics | CR/PR (n=30) | SD (n=37) | PD (n=5) |
| --- | --- | --- | --- |
| Age, median (range), years | 74.0 (59–88) | 74.0 (41–89) | 77.0 (49–85) |
| Male, n (%) | 27 (90.0) | 31 (83.8) | 5 (100.0) |
| ECOG PS 0, n (%) | 28 (93.3) | 33 (89.2) | 5 (100.0) |
| Etiology of HCC, n (%)  HBV HCV NBNC | 4 (13.3) 10 (33.3) 16 (53.3) | 3 (8.1) 12 (32.4) 22 (59.5) | 1 (20.0) 0 4 (80.0) |
| Child-Pugh score, n (%)  A5 A6 | 19 (63.3) 11 (36.7) | 24 (64.9) 13 (35.1) | 5 (100.0) 0 |
| ALBI grade, n (%)  1 2 | 12 (40.0) 18 (60.0) | 19 (51.4) 18 (48.6) | 4 (80.0) 1 (20.0) |
| AFP, median (range), ng/mL | 20.6  (3.2–193000.0)* | 8.8  (2.0–34473.0) | 8125.9  (2.0–294127.0) |
| AFP, ≥400 ng/mL, n (%) | 3 (10.0)* | 4 (10.8) | 4 (80.0) |
| Prior treatment history, n (%)  HAIC RFA, MWA Surgery Radiotherapy | 1 (3.3) 5 (16.7) 8 (26.7) 0 | 0 10 (27.0) 12 (32.4) 1 (2.7) | 0 0 3 (60.0) 0 |
| Maximum tumor diameter, median (range), cm | 4.2 (1.1–13.0) | 5.0 (1.0–11.7) | 5.1 (1.6–12.2) |
| Sum of longest diameters, median (range), cm | 5.8 (1.5–17.1) | 7.1 (1.0–21.1) | 10.1 (3.2–17.4) |
| Number of tumors, 1–5/≥6,  n (%) | 23 (76.7)/ 7 (23.3) | 24 (64.9)/ 13 (35.1) | 3 (60.0)/ 2 (40.0) |
| BCLC stage B up-to-eleven criteria (in/out) | 23 (76.7)/ 7 (23.3) | 23 (62.2)/ 14 (37.8) | 2 (40.0)/ 3 (60.0) |

*Data was missing for one patient.

AFP, alpha-fetoprotein; ALBI, albumin-bilirubin; ECOG, Eastern Cooperative Oncology Group; HAIC, hepatic arterial infusion chemotherapy; HBV, hepatitis B virus; HCV, hepatitis C virus; MWA, microwave ablation; NBNC, non-B, non-C hepatitis; PS, performance status; RFA, radiofrequency ablation; TACE, transcatheter arterial chemoembolization.

**Supplementary Table S2.** Baseline demographic and clinical characteristics with and without curative intent therapy.

| Baseline characteristics | Curative intent therapy: Resection/ RFA (n=2) | Curative intent therapy: TACE with curative intent (n=8) | Did not receive curative intent therapy (n=64) |
| --- | --- | --- | --- |
| Age, median (range), years | 70.0 (69, 71) | 77.5 (69, 84) | 74.0 (41, 89) |
| Male, n (%) | 2 (100.0) | 7 (87.5) | 56 (87.5) |
| ECOG PS 0, n (%) | 2 (100.0) | 8 (100.0) | 58 (90.6) |
| Etiology of HCC, n (%)  HBV HCV NBNC | 0 0 2 (100.0) | 0 3 (37.5) 5 (62.5) | 8 (12.5) 20 (31.3) 36 (56.3) |
| Child-Pugh score, n (%)  A5 A6 | 2 (100.0) 0 | 5 (62.5) 3 (37.5) | 42 (65.6) 22 (34.4) |
| ALBI Grade, n (%)  1 2 | 1 (50.0) 1 (50.0) | 4 (50.0) 4 (50.0) | 30 (46.9) 34 (53.1) |
| AFP, median (range), ng/mL | 193000.0  (193000.0–193000.0)* | 20.3  (2.0–391.0) | 19.0  (2.0–294127.0) |
| AFP ≥400 ng/mL, n (%) | 1 (50.0)* | 0 (0, 0) | 11 (17.2) |
| Prior treatment history, n (%)  HAIC RFA, MWA Surgery Radiotherapy | 0 0 0 0 | 1 (12.5) 1 (12.5) 1 (12.5) 0 | 1 (1.6) 14 (21.9) 22 (34.4) 2 (3.1) |
| Maximum tumor diameter, median (range), cm | 11.7 (10.9–12.4) | 5.5 (2.3–7.2) | 4.4 (1.0–13.0) |
| Sum of longest diameters, median (range), cm | 15.1 (13.0–17.1) | 9.1 (3.8–10.8) | 6.4 (1.0–21.1) |
| Number of tumors 1-5 /≥ 6, n (%) | 1 (50.0)/1 (50.0) | 4 (50.0)/4 (50.0) | 46 (71.9)/18 (28.1) |
| BCLC stage B up-to-eleven criteria (in/out) | 0/2 (100.0) | 5 (62.5)/3 (37.5) | 44 (68.8)/20 (31.3) |
| Best overall response prior to curative intent therapy (mRECIST), n (%)  CR PR SD PD NE | 1 (50.0) 1 (50.0) 0 0 0 | 0 5 (62.5) 3 (37.5) 0 0 | 8 (12.5) 15 (23.4) 34 (53.1) 5 (7.8) 2 (3.1) |

*Data was missing for one patient.

AFP, alpha-fetoprotein; ALBI, albumin-bilirubin; ECOG, Eastern Cooperative Oncology Group; CR, complete response; HAIC, hepatic arterial infusion chemotherapy; HBV, hepatitis B virus; HCV, hepatitis C virus; MWA, microwave ablation; NBNC, non-B, non-C hepatitis; NE, not evaluable; PR, partial response; PS, performance status; RFA, radiofrequency ablation; SD, stable disease; TACE, transcatheter arterial chemoembolization.

**Supplementary Figure S1. Overall survival in patients with objective response (CR/PR) vs SD vs PD per mRECIST (A) without landmarks and (B) with 6-month landmarks.**

**
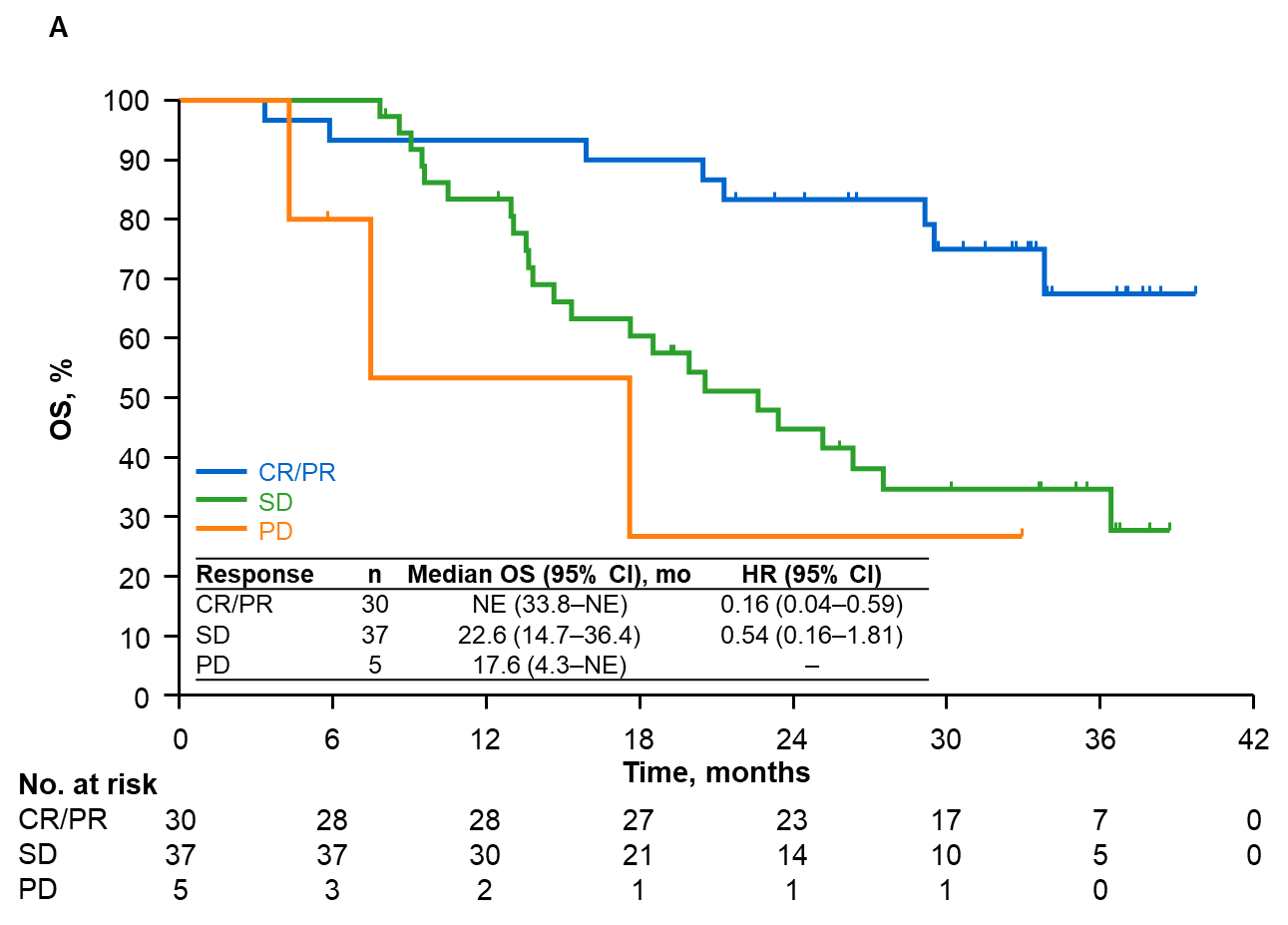
**

**
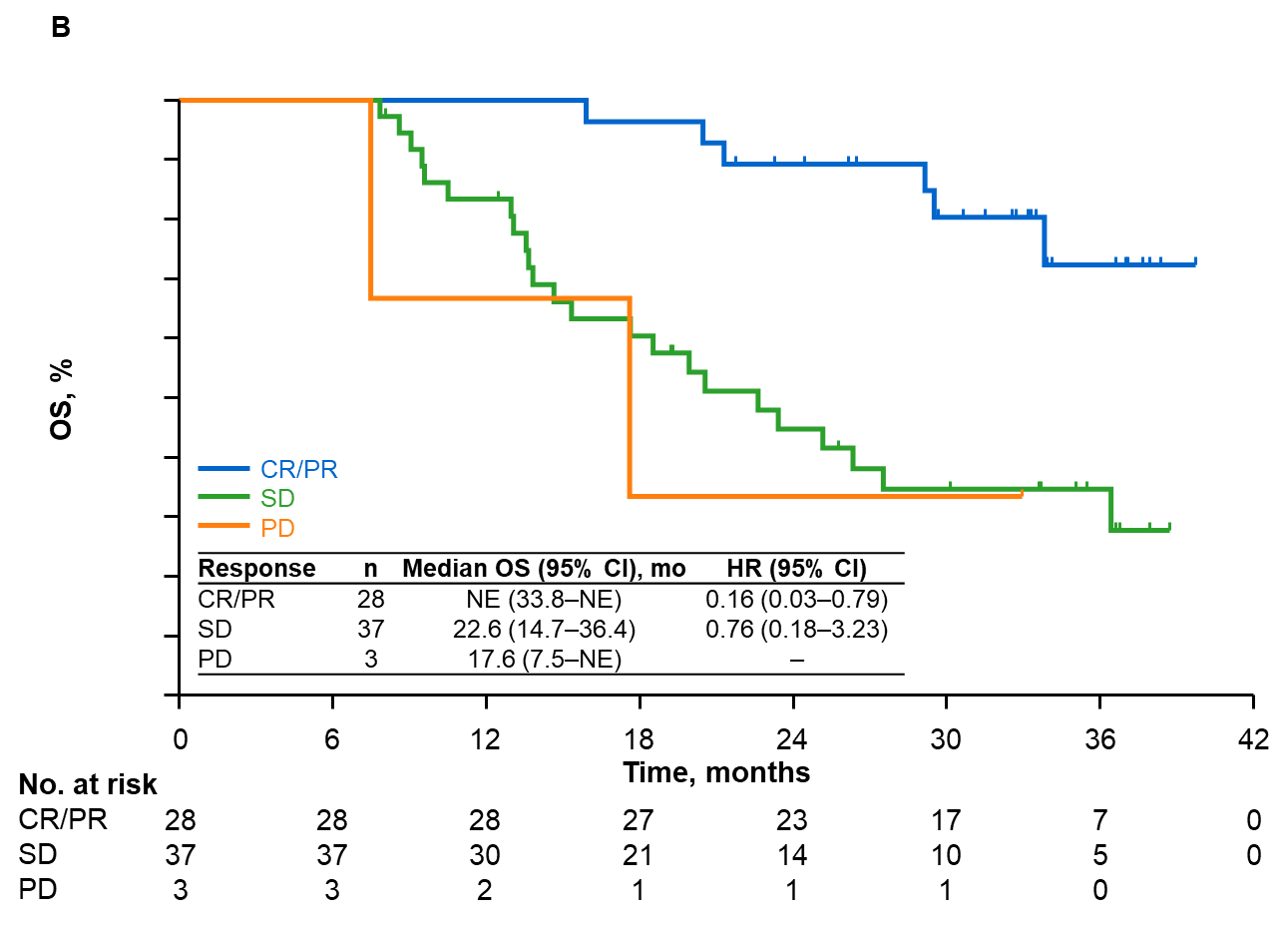
**

CR, complete response; NE, not estimable; OS, overall survival; PD, progressive disease; PR, partial response; SD, stable disease.

**Supplementary Figure S2. Overall survival in patients who received subsequent curative intent therapy vs those who did not (A) without landmarks and (B) with 6-month landmarks.**

**
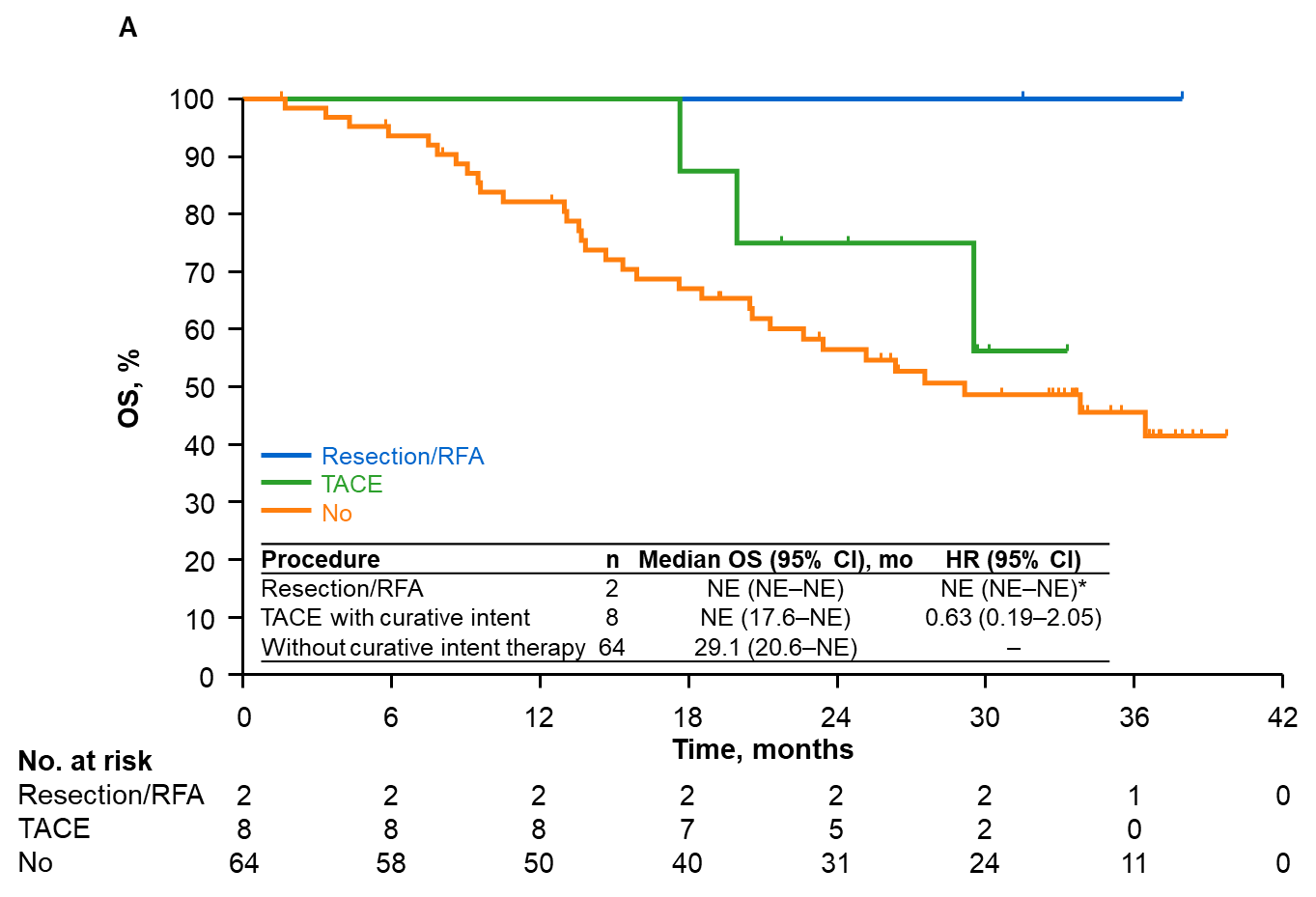
**

**
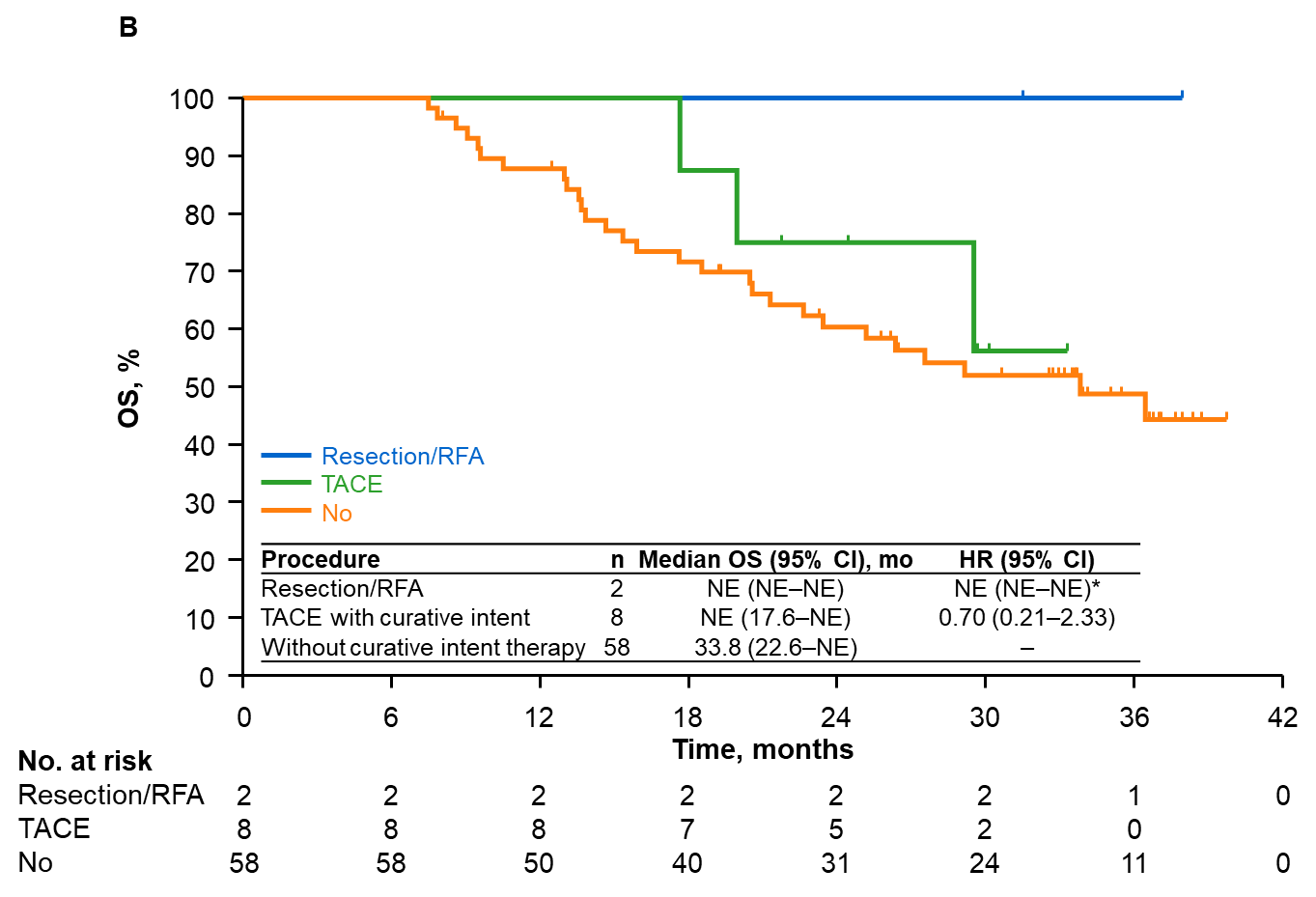
**

*The hazard ratio for the resection/RFA group was not estimable because no events occurred in this subgroup (n=2).

NE, not estimable; OS, overall survival; RFA, radiofrequency ablation; TACE, transarterial chemoembolization.

**Supplementary Figure S3. Overall survival in patients who received subsequent curative intent therapy vs those who did not, stratified by best overall response (A) without landmarks and (B) with 6-month landmarks.**


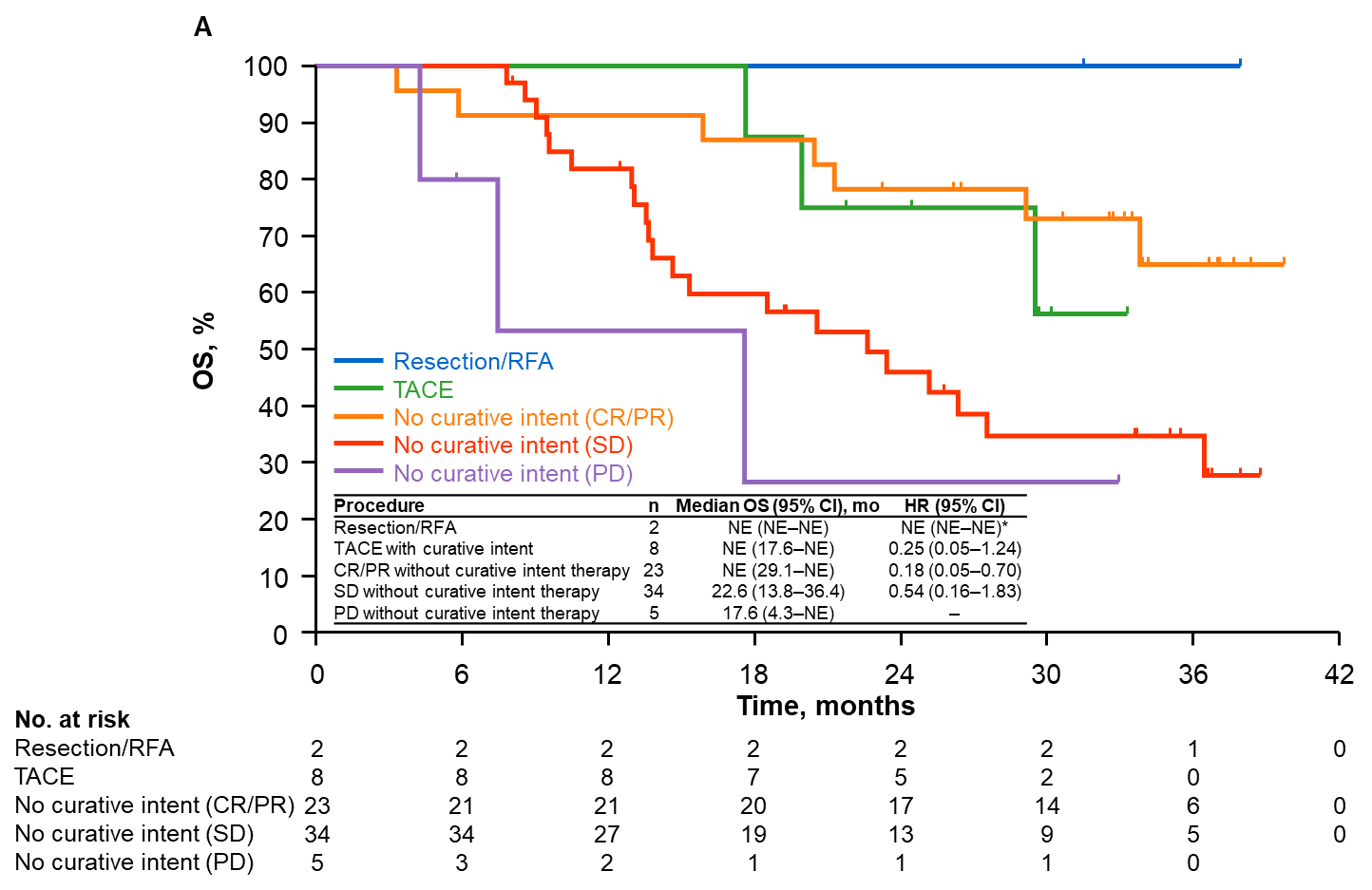


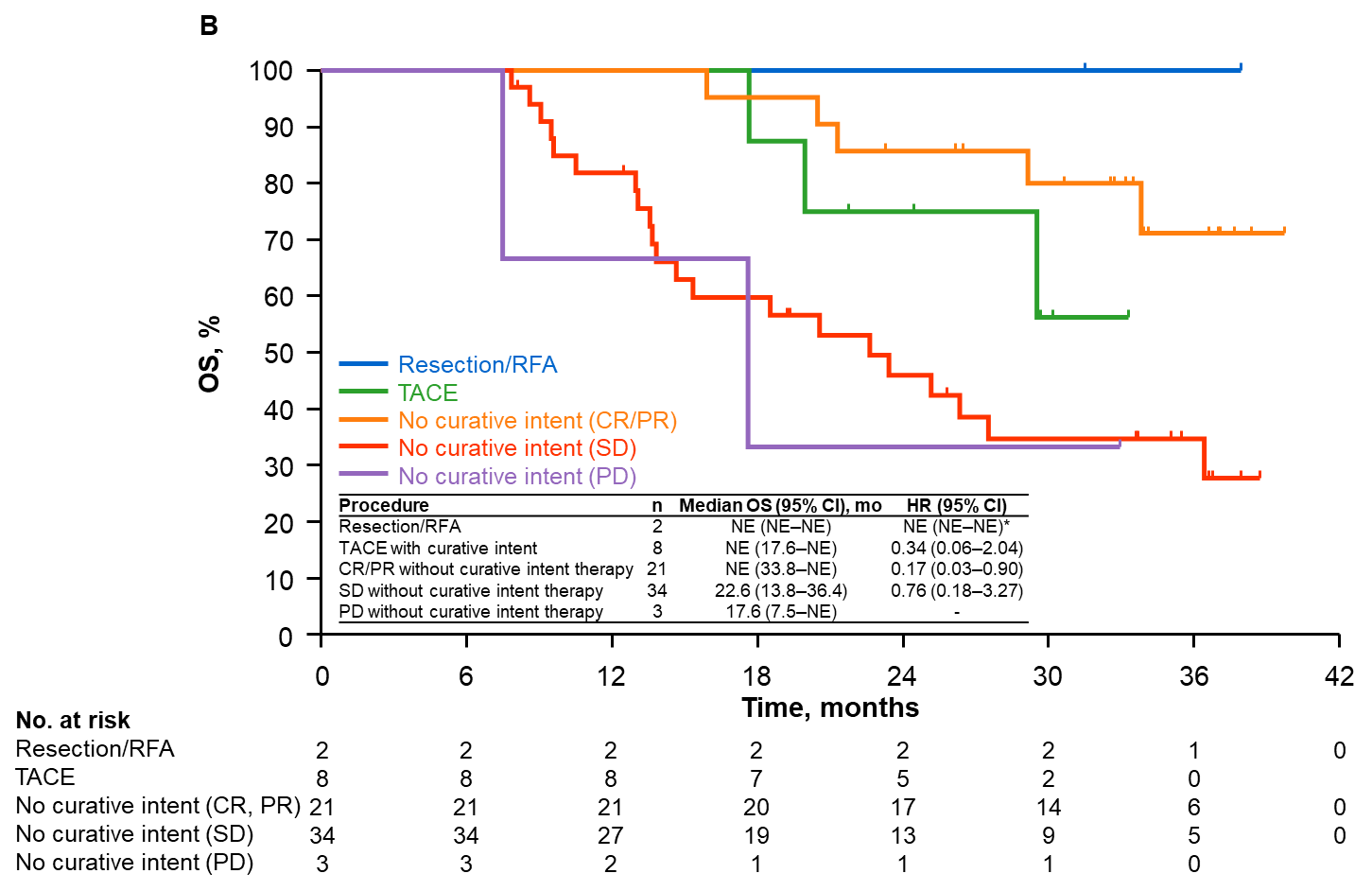

*The hazard ratio for the resection/RFA group was not estimable because no events occurred in this subgroup (n=2).

CR, complete response; NE, not estimable; OS, overall survival; PD, progressive disease; PR, partial response; RFA, radiofrequency ablation; SD, stable disease; TACE, transarterial chemoembolization.
